# PERSPECTIVES ON THE RISK FACTORS ASSOCIATED WITH MISSING INCIDENTS IN PERSONS LIVING WITH DEMENTIA

**DOI:** 10.1093/geroni/igac059.2379

**Published:** 2022-12-20

**Authors:** Christine Daum, Hector Perez, Antonio Miguel-Cruz, Elyse Letts, Emily Rutledge, Lili Liu

**Affiliations:** University of Waterloo, Waterloo, Ontario, Canada; University of Waterloo, Waterloo, Ontario, Canada; University of Alberta, Edmonton, Alberta, Canada; University of Waterloo, Waterloo, Ontario, Canada; University of Waterloo, Waterloo, Ontario, Canada; University of Waterloo, Waterloo, Ontario, Canada

## Abstract

Persons living with dementia are at higher risk of getting lost and going missing. The adverse outcomes of missing incidents are stressful for persons living with dementia and those who care for them. This study aimed to identify and describe the perspectives of persons with dementia, caregivers and community support organizations on risk factors. Generic qualitative description informed our methods. We conducted 30 virtual interviews with persons who live with dementia, professional and family caregivers and community support organization representatives. We used a card sort to elicit and describe perspectives on the importance of 27 risk factors commonly associated with missing incidents in persons living with dementia. Interviews were digitally recorded, transcribed verbatim, and subjected to content analysis to determine the presence of relevant words, themes, and concepts. Participants reported multiple experiences of a person going missing, impressions, and suggested relationships between factors such as environmental contexts. The most critical risk factors associated with getting lost and going missing were cognitive impairment, unmet needs, and inadequate concentration of services and resources. In contrast, race, education, and gender were perceived as unimportant pertaining to risk factors related to missing incidents in persons living with dementia. An understanding of the perceived importance of risks associated with missing incidents enhances a person-centered approach to addressing unmet needs, services and resources that balances quality of life with maintaining safety.

